# Does Mothers' Awareness of Health and Nutrition Matter? A Case Study of Child Malnutrition in Marginalized Rural Community of Punjab, Pakistan

**DOI:** 10.3389/fpubh.2022.792164

**Published:** 2022-02-08

**Authors:** Muhammad Shahid, Yang Cao, Farooq Ahmed, Saqlain Raza, Jing Guo, Najma Iqbal Malik, Umara Rauf, Madeeha Gohar Qureshi, Rafit Saheed, Rohma Maryam

**Affiliations:** ^1^School of Insurance and Economics, University of International Business and Economics (UIBE), Beijing, China; ^2^Department of Anthropology, Quaid-I-Azam University, Islamabad, Pakistan; ^3^Department of Anthropology, University of Washington, Seattle, WA, United States; ^4^Respiratory Care Department, College of Applied Medical Sciences in Jubail, Imam Abdulrahman Bin Faisal University, Al Jubail, Saudi Arabia; ^5^Department of Health Policy and Management, School of Public Health, Peking University, Beijing, China; ^6^Department of Psychology, University of Sargodha, Sargodha, Pakistan; ^7^Department of Psychology, Government College Women University Sialkot, Sialkot, Pakistan; ^8^Department of Economics, Pakistan Institute of Development Economics (PIDE), Islamabad, Pakistan; ^9^School of Public Policy, Pakistan Institute of Development Economics (PIDE), Islamabad, Pakistan; ^10^Department of Biotechnology, Quaid-I-Azam University, Islamabad, Pakistan

**Keywords:** health and nutritional awareness, household deprivation, malnutrition, Pakistan, odds ratio

## Abstract

Maternal nutritional awareness might reduce the risk of malnutrition in children. This study assesses the impact of mothers' nutritional and health awareness (MNHA) on the nutritional status of pre-school children in rural South Punjab. Using a proportionate purposive simple random sampling technique we collect data with the help of a self-administered questionnaire on height, age, the weight of children, and socio-economic profile from 384 rural households in one of the marginalized districts of Punjab. The study applied the binary logistic regression model to compute the probability of malnutrition. The results indicate that malnutrition was high in the district (the prevalence rate for underweight is 46.1%, for stunting 34.83%, and for wasting is 15.49%). Around 91.84% of malnourished children belonged to the low MNHA category compared to medium (5.61%) and high (2.55%) MNHA categories. The results further show that the prevalence of moderate and severe stunting, wasting, and underweight in low MNHA categories was much higher with large differences compared to both medium and high MNHA categories. The binary logistic regression results depict that, across the household deprivation index (HDS), the odds of a child becoming malnourished were lower in households HDS-2 category (OR = 0.02, 95% CI: 0.01–0.89), and odds were also lower in households HDS-3 category (OR = 0.001, 95% CI: 0.001–0.16). Similarly, across the scores of MNHA index, the odds of malnutrition were lower among the children of those mothers who had medium MNHA (OR = 0.04, 95% CI: 0.002–1.24), and also the probability of child malnutrition was lower among the children of mothers who had high MNHA (OR = 0.008, 95% CI: 0.002–0.29). The study urges that well-resourced, targeted, and coordinated health and nutritional education and awareness programs are required to tackle malnutrition.

## Introduction

Malnutrition is a multifaceted problem as it is one of the most significant public health concerns of the Pakistani government. Malnutrition is still high in Pakistan, about 38% of under 5-year children are stunted, 23% are under-weight and 7% are wasted (PDHS, 2017–18). It is of vast interest as to why malnutrition remains high in Pakistan. In the light of previous researches, it is fact that child malnutrition cannot be tackled without understanding its accurate causes. Most of the literature on causes of malnutrition depicts that household poor socio-economic/wealth status is a main unseen cause behind child malnutrition (1–6). It is acknowledged that mothers' education (formal and on nutrition) is an important factor even after controlling wealth or socioeconomic variables (1). Previous Literature highlighted that mothers' education is highly associated with child development (7–10). It is argued that child care especially feeding, food serving in-home, medical needs against illness mostly depend on mothers, so educated mothers can raise their children more healthily. Mothers having nutrition knowledge can be healthily raised their children by providing a balanced diet to them (11, 12).

Poor nutritional awareness and education of mothers have been identified as one of the major causes of child malnutrition in many studies (13–17). There is a consensus that low nutritional awareness in mothers and household socioeconomic deprivation are the main risk factors of child malnutrition. To find the answer, whether mothers' poor nutritional and health awareness in the socioeconomically deprived segment of society is contributing to children's nutritional status or not is the main concern of the research. First of all, there are limited researches on the impact of MNHA on child malnutrition, particularly in those women who getting education informally in Pakistan. Secondly, in the context of Pakistan, it remains still unclear the nature of the relationship between the nutritional knowledge of illiterate mothers and their children's nutritional status. Thirdly, the researchers in Pakistan assessed the relationship between mothers' education (school or formal education) with malnutrition and mothers' nutritional awareness in the case of only food items. It remains uncertain what type of information and education a mother should have about the health and nutrition of their children. The mother must be given enough awareness not only regarding the type of food items given to the child but also the knowledge of screening malnutrition and diseases for protection. This present study covers these gaps.

This study constructed a mother's nutritional and health awareness index (MNHA) based on previous literature considering all the dimensions and themes regarding health and nutritional education and awareness. The research identified five components for the measurement of MNHA; 1- Immunization, 2- Food diversity, 3- Fertility, 4- Diagnosis of malnutrition, 5- awareness of nutrition. If the mother is aware of the above five dimensions of the MNHA then it could be hypothesized that malnutrition can be reduced to a large extent along with better socio-economic status. So, the question of this research is whether MNHA enlarges the role of socioeconomic status in malnutrition reduction or not. District Rahimyar Khan is one of the largest districts of Southern Punjab which shows a true representation of rural Punjab, having about 78.5% population living in rural areas (18). Most of its population is living with poor socio-economic status facing difficulties to meet their ends. Rahimyar Khan is included among one of the high malnutrition districts in the Punjab province with a very low literacy rate (19). In the district, child mortalities are high especially in rural areas. According to Punjab Development Statistics, this district stood fourth in Punjab in high rates of infants and under-five mortalities (20). Hence to see the impact of MNHA in socioeconomically marginalized rural society in Punjab, we selected the Rahimyar Khan district in Punjab.

## Conceptual Framework

**Figure 1:**
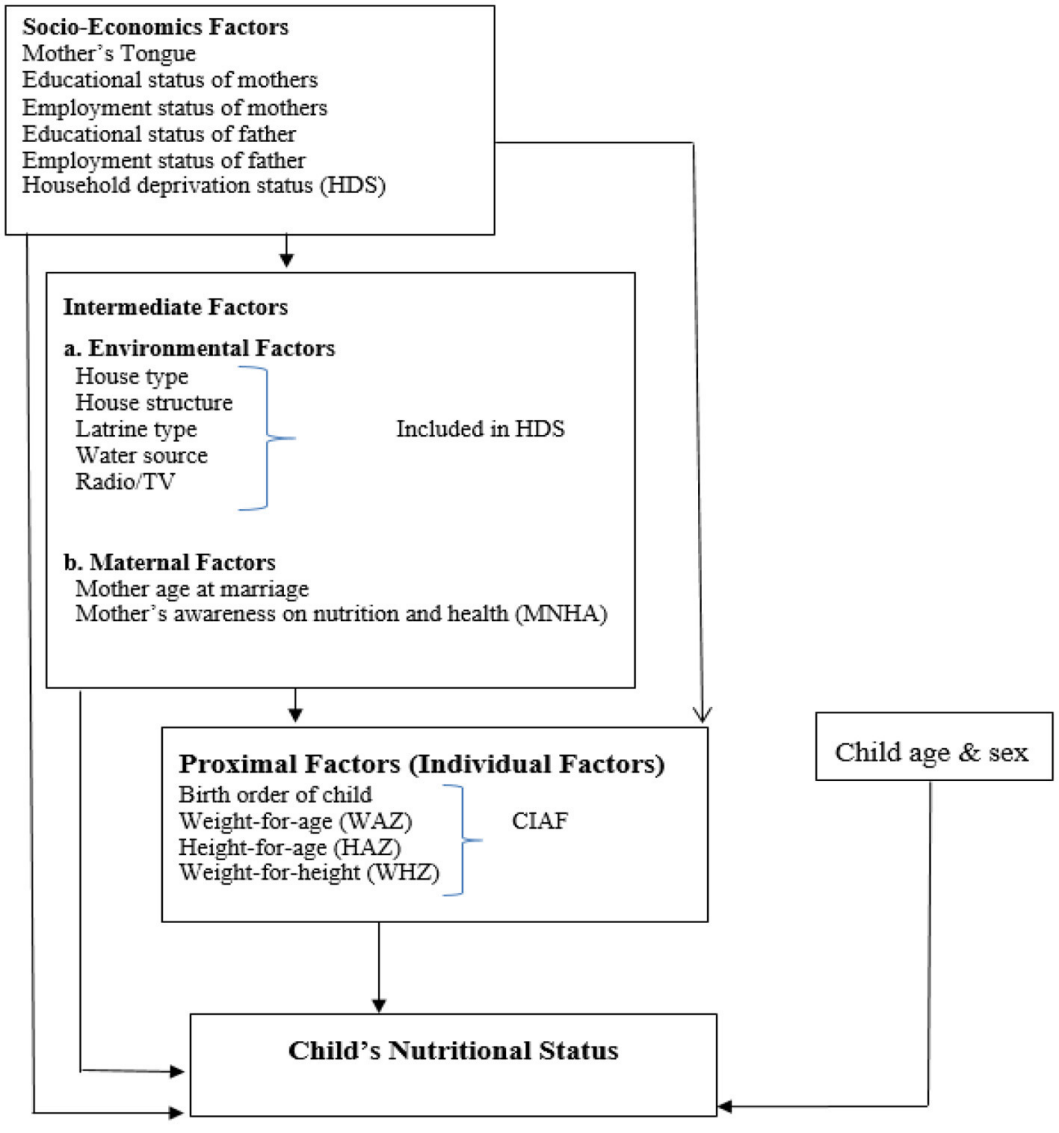
Conceptual framework for determinants of child malnutrition. Victora et al. (21).

This study followed the conceptual framework of Victora (21). The distribution of variables in this framework is in three group types: socio-economic reasons, intermediate factors containing maternal and environmental issues, and proximal or individual aspects. In short, pre-school children's nutritional status may be affected by these factors (21). The conceptual framework of the study is given below in (Figure 1).

## Methods

### Study Area, Sampling, and Data Collection

This study collected the primary data through a self-administered questionnaire using a proportionate purposive simple random sampling technique from households in rural areas of district Rahimyar Khan. The sample was allocated proportionately among four sub-districts [(1)-KhanPur, (2)-Liaquatpur, (3)-Rahimyar Khan, and (4)-Sadiqabad]. The design of the sample was established based on the probability proportional to the size (PPS) in all four tehsils of district Rahimyar Khan. The sampling frame consisted of all rural households in the district. The first stage was a stratified random sampling of rural clusters [village; also called Union Councils (UCs) in every tehsil/subdivision]. The rural households for the survey were chosen randomly through the lady health worker register record.

During the survey, if there is more than one family in one premises or joint families in one house, then the study considered them nuclear if they make food independently. The anthropometric measurements were taken by lady health workers as they were trained enough before assigning the task of anthropometric measurements. We used MUAC tape, a weight machine, measurement tape for collecting data on the height, weight, along with the age of children and their mothers.

Data was gathered during 3 months in the study area from November 2017 to January 2018. After the approval of the district health officer, mothers and their close families were informed in local languages (Punjabi and Saraiki) through lady health workers about the nature of the study 1 week before to seek their verbal consent and willingness to participate in the study. All mothers from 384 households agreed to participate voluntarily in the study, and mothers gave their verbal consent during the pre-interview meeting. Written consent was not sought as the majority of the mothers (74%) had no formal education and they were also reluctant due to their cultural bounds. The sample size that came to be (*n* = 384 households) using Raosoft calculator, keeping the confidence interval at 5% and confidence level as 95%. The detail about sample size calculation is given below:


$$\begin{array}{l} \text{Sample size}={\text{Z}}^{2}\;*\;\left(\text{p}\right)\;*\;\left(1-\text{p}\right)/\text{c}{\;}^{2} \end{array}$$

Where:

Z = Z value (e.g., 1.96 for 95% confidence level).
p = Percentage picking a choice, expressed as decimal (0.5 used for sample size needed).
c = Confidence interval, expressed as decimal (e.g., 0.04 = ± 4).
Sample size = (1.96)^2^^*^(0.5) ^*^ (1-0.5) / (0.05)^2^ = 384.16 = 384.

The detail about the proportional allocation of n=384 in twelve Union Councils is given below in (Table 1):


$$\begin{array}{l} \text{NI}=\text{n }*\text{ Ni}/\text{N} \end{array}$$

The formula for each UC sample calculation = Population of UC 1, 2, 3/total Population of 3 unions councils^*^sample size of tehsil.

NI = Number of sampled respondents in each union council.
I = Number of UCs in study area i.e., 1, 2, 3…., 12.
n = Total sample size.

**Table 1:** Proportional allocation of sample size (*n* = 384) distribution from Tehsils to Union Councils.

**Tehsil name with allocated sample**	**Name of UCs with population**	**Approximate sample from Tehsil to Union Council**
Khanpur	Bagho Bahar (Pop = 24,349)	N1 = No. of respondent's in UC1 = 24349/90811*96 = **26**
**96**	Azeem Shah (Pop = 32,876)	N2 = No. of respondent's in UC2 = 32876/90811*96 = **34**
	Kotla Pathan (Pop = 33,586)	N3 = No. of respondent's in UC3 = 33586/90811*96 = **36**
Liaquatpur	Ghooka (Pop = 39,367)	N4 = No. of respondent's in UC4 = 39367/126573*81 = **25**
**81**	Shadani (Pop = 40,990)	N5 = No. of respondent's in UC5 = 40990/126573*81 = **26**
	Trinda Gurgaij (Pop = 46,216)	N6 = No. of respondent's in UC6 = 46216/126573*81 = **30**
Rahimyar Khans	Bahishti (Pop = 32,870)	N7 = No. of respondent's in UC7 = 32870/111793*115 = **34**
**115**	Sonak (Pop = 45,423)	N8 = No. of respondent's in UC8 = 45423/111793*115 = **46**
	Chak No. 84/P (Pop = 33,500)	N9 = No. of respondent's in UC9 = 33500/111793*115 = **35**
Sadiqabad	Kot Sanger Khan (Pop = 31,543)	N10 = No. of respondent's in UC10 = 31543/88105*92 = **33**
**92**	Muhammad Pur (Pop = 31,269)	N11 = No. of respondent's in UC11 = 31269/88105*92 = **32**
	Roshan Bhet (Pop = 25,293)	N12 = No. of respondent's in UC12 = 25293/88105*92 = **27**
**Total** **=** **384**	**Union Councils** **=** **12**	***n*** **=** **384**

*Authors. Bold values shows the sample size in tahsils and each union councils*.

### Outcome Variable

The study dichotomized the dependent variable (CIAF) into two categories: “1” if a child is malnourished otherwise use “0” if a child is not malnourished. According to CIAF classification, children are divided into seven groups which are as follows: A: no failure, B: stunted only, C: wasting only, D: underweight only, E: stunted and underweight, F: wasting and underweight, and last is G: stunting, wasting, and underweight. CIAF estimates the overall presence of malnutrition in children. The total measure of child malnutrition prevalence is calculated by combinations of all groups except group A. Outcome variable (CIAF) was based on three indices; HAZ, WHZ, and WAZ which is stated by WHO child growth standards guidelines (2009) followed by anthropometric measurements (22).

### Independent Variables

This study follows the conceptual framework of Victoria for the choice of possible determinants of child malnutrition (21). The independent variables for the analysis included in the study were the gender (male, female), age (0–5 years), birth order of child (1, 2–3, 4–5, and ≥6), educational status of mother and father (illiterate, primary, middle, matric, intermediate and higher), mother working status (working or not working), father working status (Government job, private job, own business, daily wages/labor, and agriculture), mother's tongue or ethnicity (Saraiki or Punjabi), Mother's age at marriage (<18, 18–25, and >25 years), household deprivation status (HDS-1, HDS-2, HDS-3), and MNHA (Low, Medium, High).

This study used the household deprivation status index of Srinivasan and Mohanty (23, 24). In the HDS index, 6 variables are used which are as follows: (1)- household has a mud house type or have cemented type, (2)- some landholding by the household or not, (3)- electricity facility is available in the house or not, (4)- within the residence or household is drinking facility available or not, (5)- any one member in the household is literate or not, (6)- keeping T.V, radio or newspaper in the house or not. The variables are in binary form. Adding of these six variables shows the total scores and the range of scores is 0–6. Those having none of any items from six possessions or have only 1 or 2 items, includes in HDS-1 and are called “moderate deprivation (MD)” that shows deprived segments of the population. “Just above the deprivation (JAD)” indicates those which have 3 possessions included in HDS-2. In HDS-3, those who have 4 or 6 items, it indicates “well-above the deprivation (WAD).” The HDS is not a direct measure for economic conditions of the household like total expenditure, per capita income, or living standard index, however, it extends the measure to depict the household above of the three dimensions as deprived (23, 24).

### Development of Health and Nutrition Awareness Index

We developed the MNHA index based on previous studies (11–15, 25–40) searched from google scholar and Pubmed. We considered only those studies which tested the relationship between mothers' nutritional and health-related attitude/awareness and malnutrition. The main focus remained on studies that used mothers' nutritional and health-related education/attitude/awareness variables or indexes, and further, they related with malnutrition of child. We found ~25 articles specifically on the relationship of mothers' nutritional and health awareness with malnutrition. Following keywords for literature search were used: (1) nutritional awareness and malnutrition; (2) nutrition education and malnutrition; (3) health knowledge and malnutrition; (4) mothers health awareness and malnutrition; (5) mothers' nutritional knowledge and malnutrition; (6) mothers nutritional and health education and malnutrition; (7) mothers nutrition and health awareness and malnutrition.

Most of the studies were concluding that although general formal education of mothers is an important factor the mother's nutritional or health-related education/attitude/awareness has much significant role in reducing malnutrition in children. During literature on different mothers' nutritional and health awareness/attitude studies, the authors noticed that information in most of the researches in their index or mothers nutritional awareness/attitude variables were related to food consumption/food diversity, diagnosis of malnutrition, or malnutrition-related care, immunization and infection-related information, fertility or birth intervals link with adverse health, and awareness about nutrition. After searching relevant literature we discussed it with a few experts of the field for validation. In their opinion, the awareness tool must be easy to understand, short, and simple for local mothers. Therefore, the study combined only five basic questions to build the MNHA index covering the most important aspects of health and nutrition.

The study developed the MHNA index, which is a key independent variable of this study. This index consists of five questions regarding attitudes and awareness of mothers on health and nutrition which are as follows: Are you aware of the immunization programs? Does complete immunization save children from infections? Do you feel that milk/egg/meat/vegetables are essential for that food item of children? Do you feel that continuous birth interval adversely affects a child's overall health? Do you have any idea about the underweight problem of children? Have you noticed this in the case of your children? Do you know what makes the children weak?

The index is in the binary form; if the answer of the mother to the question is “Yes” it assigns a value equal to 1 otherwise 0 if the answer is “No”. Further, this index is categorized into three groups; low, medium, and high awareness of mothers on nutrition. The value range for the low MNHA group is from 0 to 2; for the medium MNHA, the value is 3 only, and for the higher MNHA group the range of value is 4–6. The high value shows that mothers have good knowledge of child's health and nutrition.

### Statistical Analysis

The surveyed data was in three files. Household records and children's record files were merged for analysis. Before testing the relationship between CIAF and explanatory variables, the data were cleaned by all outliers. From the data, the Z-scores that were outside the WHO flags were also skipped. For analysis study included 316 children while 201 children Skipped from analysis out of a total of 517 under-five children because of over range (<5 and >+5). Cross-tabulations were measured as descriptive statistics to get the percentage of the occurrence of CIAF (malnutrition) concerning each explanatory variable. The logistic regression technique was applied to assess the influence of socioeconomic factors on malnutrition. Analysis of data was taken on statistical package STATA 15.

### Binary Logistic Regression

To evaluate the connection between MNHA and children nutritional status this study employs the logistic regression method which measures the probability of malnutrition in two ways (“1” if a child is malnourished otherwise use “0” if a child is not malnourished), by hypnotizing that malnutrition of children depends on many proximate factors including household socio-economic, maternal and child characteristics. Malnutrition was assessed by the CIAF, a measure that is based on wasting, stunting, and being underweight. To assess the association among malnutrition and a set of explanatory variables binary logistic regression was used as this regression estimates the probability of outcome variable (CIAF) conditioned on many proximate indicators. The model specification and reduced form of binary logistic regression are shown below:


$$\begin{array}{l} P\;(Yi=1|X_{\text{1i}},X_{\text{2i}}\ldots ,Xkn)\\ \;=F\;({\text{β}}_{0}+{\text{β}}_{1}X_{\text{1i}}+{\text{β}}_{2}X_{\text{2i}}+\ldots .+\;\beta_{\text{n}}X_{\text{kn}}) \end{array}$$

CIAFi = f (Individual factors, Environmental factors, Maternal Factors, Socio-Economic Factors, εCIAF).

In this equation, y_i_ denote indicators of child malnutrition i as dependent variable (CIAF); X shows explanatory variables; β's are coefficients of interest, which explain the degree of association with dependent variable CIAF; εCIAF is a random error assumed with covariates in the reduced form which shows nutrition outcome function can be uncorrelated. Here Y is a binary outcome variable (CIAF) and (Yi = 1) implies that the child is malnourished, and (Yi = 0) represents child is not malnourished, X = (X_1i_, X_2i_…, Xkn) are independent variables, and xi is the observed value of the independent variable for observation i.

## Results

### Profile of the Respondents

Most of the mothers (76.53%) were without any formal education in the district and 84.18% among them belonged to the 18–25 years age group at marriage. Around 58% of households had an annual income of <50,000 Pakistani Rupees, while 26% of households' annual income was <100,000 Pakistani Rupees. Around 91% of households belonged to the HDS-1 (2.5%) and HDS-2 (88.78%) category-the most deprived segment of the society. Around 79% head of the households (fathers) were daily wagers, laborers, or agricultural employment. Around 92% of mothers were poor in MNHA. A total of 517 Under-Five children were sampled for the study, out of which 286 (56%) were male children and 231 (44%) were female children.

### Descriptive Statistics

The prevalence of malnutrition was very high in the district (the prevalence rate for underweight is 46.1%, for stunting 34.83%, and for wasting is 15.49%). Table 2 indicated the percentage of the occurrence of CIAF in a child concerning different socio-economic factors. The descriptive results in Table 2 showed that 47.45% male and 52.55% female children were malnourished. Around 76.53% of malnourished children belonged to those households where mothers had no education, while 79.08% of malnourished children belonged to those houses in which fathers were daily wagers/labor or engaged in agricultural work. Mothers who were not employed had 98.47% of malnourished children and the households belonging to the HDS-2 category which is the deprived segment of the society had 88.78% malnourished children in their houses. This suggests that child malnourishment is due to MNHA, as well as, unemployment and poverty. Mothers who have low MNHA scores had 91.84%, malnourished children. The descriptive results showed that malnutrition prevalence rates were high among those households who were deprived and their females were mostly illiterate and their mothers had low MNHA scores. Around 87.24% of malnourished children were Saraiki ethnic group.

**Table 2:** Descriptive analysis describing the association between different socioeconomic characteristics over CIAF (child malnutrition) (*N* = 316).

**Variables**	**Categories**	**Frequencies**	**Percentages**	***P*-Values**
Sex of child	Male	93	47.45	0.140
	Female	103	52.55	
Age of child	0–12	19	9.69	0.000^***^
(in months)	13–24	26	13.27	
	25–36	60	30.61	
	37–48	51	26.02	
	49–60	40	20.41	
Birth order number	Birth order 1	52	26.53	0.079^*^
	2 or 3	79	40.31	
	4 or 5	41	20.92	
	6 or above	24	12.24	
Mother's age	<18 years	22	11.22	0.559
at marriage	18–25 years	165	84.18	
	>25 years	9	4.59	
Mother's	No education	150	76.53	0.788
education	Primary	22	11.22	
	Middle	14	7.14	
	Matric	6	3.06	
	FA & Higher	4	2.04	
Father's education	No education	135	68.88	0.403
	Primary	35	17.86	
	Middle	7	3.57	
	Matric	16	8.16	
	FA & Higher	3	1.53	
Mother's working	Working	3	1.53	0.012^***^
status	Not-working	193	98.47	
Father's working	Govt. job	8	4.08	0.133
status	Private job	8	4.08	
	Own business	25	12.76	
	Daily wages/Labor & Agriculture	155	79.08	
Mother's	Punjabi	25	12.76	0.114
tongue/ethnicity	Saraiki	171	87.24	
Household	HDS-1	5	2.55	0.084^*^
deprivation status	HDS-2	174	88.78	
	HDS-3	17	8.67	
Nutritional and	Low	180	91.84	0.008^***^
health awareness	Medium	11	5.61	
of mothers	High	5	2.55	

*Authors' estimation [Significance level:*

*** *if P < 0.01*,

** *if P < 0.05*,

* *if P < 0.1]*.

Table 3 illustrates the association among all three indices of child nutritional status with MNHA. According to the weight for age classification (underweight), moderate underweight in all the three groups which are Low MNHA, medium MNHA, and high MNHA was 22.68, 2.23, and 0%, while the severe underweight prevalence in all three groups in pre-school children was 43.12, 1.86, and 1.11%, respectively. For classification of height for age (stunting), moderate stunting prevalence in all the three groups was 32.58, 1.87, and 0.37%, while this rate of prevalence for severe stunting in all three groups among pre-school children was 32.58, 0.76, and 1.49%, respectively. Furthermore, in weight for height classification (wasting), prevalence rates of moderate wasting in all the three groups were 23.94, 4.22, and 1.41%, while in case of severe wasting in all three groups in pre-school children was 12.68, 2.82, and 0%, respectively. The moderate and severe underweight, stunting, and wasting prevalence rates in the low MNHA category were much higher compared to medium and high MNHA categories.

**Table 3:** Descriptive analysis illustrating the association between mother's nutritional and health awareness (MNHA) and anthropometric indicators.

**Indicators**	**Mother's nutritional and health awareness**				
	**Categories**	**Normal**	**Moderate**	**Severe**	**Total**
Underweight	MNHL-1 (Low)	71 (26.39%)	61 (22.68%)	116 (43.12%)	248 (92.19%)
	MNHL-2 (Medium)	2 (0.74%)	6 (2.23%)	5 (1.86%)	13 (4.83%)
	MNHL-3 (High)	5 (1.86%)	0 (0%)	3 (1.11%)	8 (2.97%)
	**Total**	**78 (29%)**	**67 (24.91%)**	**46.10 (24.91%)**	**269 (100%)**
Stunting	MNHL-1 (Low)	69 (25.84%)	87 (32.58%)	87 (32.58%)	243 (91.02%)
	MNHL-2 (Medium)	4 (1.49%)	5 (1.87%)	2 (0.76%)	11 (4.12%)
	MNHL-3 (High)	8 (3%)	1 (0.37%)	4 (1.49%)	13 (4.86%)
	**Total**	**81 (30.34%)**	**93 (34.84%)**	**93 (34.83%)**	**267 (100%)**
Wasting	MNHL-1 (Low)	31 (43.66%)	17 (23.94%)	9 (12.68%)	57 (80.28%)
	MNHL-2 (Medium)	6 (8.45%)	3 (4.22%)	2 (2.82%)	11 (15.49%)
	MNHL-3 (High)	2 (2.82%)	1 (1.41%)	0 (0%)	3 (4.23%)
	**Total**	**39 (54.93%)**	**21 (29.58%)**	**11 (15.49%)**	**71 (100)**

*Authors' estimation. Bold values shows the total frequency and percentage in cross tabulation by each section*.

### Logistic Regression Estimates

The logistic regression estimates for CIAF were displayed in (Table 4). The logistic results for age of children depicted that age of children from 25 to 36 months was associated with the higher odds of malnutrition among pre-school children in district Rahimyar Khan (OR = 8.68, 95% CI: 2.83–26.61). Birth order/interval of children 4–5 years was accompanied with lower likelihoods of malnourishment (OR = 0.44, 95% CI: 0.21–0.94). Mothers who were not engaged in any employment or job had higher odds of malnutrition among their under-five children (OR = 3.31, 95% CI: 0.31–35.69). Children who belonged to Saraiki families had lower chances of becoming malnourished as compared to Punjabi counterparts (OR = 0.39, 95% CI: 0.15–0.98). Across the HDS scores, the odds of children becoming malnourished were lower in the HDS-2 households' category (OR = 0.02, 95% CI: 0.01–0.89), and odds were also lower in the HDS-3 households' category (OR = 0.001, 95% CI: 0.001–0.16). Similarly, across the scores of MNHA index, the odds of malnutrition were lower among the children of those mothers who had medium MNHA (OR = 0.04, 95% CI: 0.002–1.24), and also the probability of child malnutrition was lower among the children of mothers who had high MNHA (OR = 0.008, 95% CI: 0.002–0.29).

**Table 4:** Binary logistic regression analysis results for CIAF (child malnutrition) and its correlates.

**Variables**	**Categories**	**Coefficients**	**Std. Err**.	**Odd. Ratios**	**95% CI**
Sex of child (female-reference)	Male	−0.358	0.1972	0.699	[0.402, 1.215]
Age of child (0 to 12 months-reference)	13–24 months	0.898	1.4558	2.455	[0.768, 7.848]
	25–36 months	2.1609	4.9607	8.679^***^	[2.831, 26.607]
	37–48 months	0.1272	0.5104	1.136	[0.471, 2.740]
	49–60 months	−0.1117	0.4129	0.894	[0.362, 2.211]
Birth order number (birth order 1-reference)	2 or 3	−0.1995	0.2986	0.819	[0.401, 1.674]
	4 or 5	−0.8183	0.1689	0.441^**^	[0.208, 0.934]
	6 or above	−0.0578	0.4821	0.944	[0.347, 2.568]
Mother's age at marriage (<18 years-reference)	18–25 years	−0.7169	0.2891	0.488	[0.153, 1.558]
	>25 years	1.3364	4.9544	3.805	[0.297, 48.83]
Mother's education (illiterate-reference)	Primary	−0.263	0.8542	0.769	[0.087, 6.786]
	Middle	−0.4636	0.7462	0.629	[0.062, 6.434]
	Matric	−1.5127	0.2932	0.220	[0.016, 2.989]
Father's education (illiterate-reference)	Primary	−0.2082	0.8851	0.812	[0.096, 6.876]
	Middle	0.7817	2.6304	2.185	[0.207, 23.126]
	Matric	1.7912	7.0102	5.996	[0.606, 59.293]
	FA & Higher	2.2146	14.356	9.158	[0.424, 197.75]
Mother's working status (working-reference)	Not-working	1.1969	4.0156	3.309*^*^	[0.307, 35.685]
Father's working status (govt. job-reference)	Private job	−1.1423	0.5235	0.319	[0.0128, 7.948]
	Own business	−1.969	0.2173	0.139	[0.007, 2.949]
	Daily Wage/ Labor & Agriculture	−1. 087	4.0149	2.966	[0.208, 42.123]
Mother's tongue/ethnicity (punjabi-reference)	Saraiki	−0.949	0.1834	0.387^**^	[0.153, 0.979]
Household deprivation status (HDS-1-reference)	HDS-2	−3.835	0.0409	0.022^**^	[0.005, 0.879]
	HDS-3	−6.6539	0.0032	0.001^***^	[0.001, 0.155]
Nutritional and health awareness of mothers (low MNHA-reference)	Medium	−3.2933	0.0664	0.037^**^	[0.002, 1.237]
	High	−4.7759	0.0154	0.008^***^	[0.002, 0.294]
**The overall significance of the model**					
No. of observations = 306		Prob>Chi^2^ = 0.0000			
LR Chi^2^ (26) = 80.09		Pseudo R^2^ = 0.1982			

*References: odd ratios; p-values; confidence intervals.

*Significance level:*

*** *if P < 0.01*,

** *if P < 0.05*,

* *if P < 0.1*.

*Authors' estimation*.

## Discussion

This study assessed the impact of maternal health and nutritional awareness (MNHA) along with household socioeconomic deprivation status (HDS) on child malnutrition status in a marginalized rural Punjab district of Pakistan. The results of the study depicted that the prevalence rate of underweight is 46.1%, stunting 34.83%, and wasting is 15.49%. The study findings showed that the age of a child, birth order of the child, ethnicity, and mother working status have a significant association with child malnutrition. Furthermore, the results of main policy variables highlighted that MNHA along with HDS largely contributed to child malnutrition. The study results further demonstrated that across the household deprivation index scores, the odds of a child becoming malnourished was lower in the HDS-2 households' category (OR = 0.02, 95% CI: 0.01–0.89), and odds were also lower in the HDS-3 households' category (OR = 0.001, 95% CI: 0.001–0.16). As the household deprivation in basic amenities of life reduced or in other words increased in basic amenities of life from HDS-2 to HDS-3, it reduced the probability of malnutrition in children as compared to HDS-1 (as HDS-1 shows that household has nothing or one or two basic amenities of life).

The results showed that in low MNHA categories the stunting, wasting, and underweight prevalence rates were much higher compared to both medium and high MNHA categories. The logistic regression depicted that, across the scores of MNHA, the odds of malnutrition were lower among the children of those mothers who had medium MNHA (OR = 0.04, 95% CI: 0.002–1.24), and also the probability of child malnutrition was lower among the children of mothers who had high MNHA (OR = 0.008, 95% CI: 0.002–0.29). Childcare depends on the mother's knowledge and education, especially on health and nutrition. Mother takes better nutritional care of a child if she was more aware of signs and causes of nutritional deficiency and further requirements of nutrition rather than the educated mothers.

Studies in Asia depicted MNHA as a curtailed indicator for child malnutrition. The study in rural Bengal endorsed that if parents showed nutritional ignorance, the frequency of malnutrition among their children remained high (25). A study in Indonesia established a maternal nutritional knowledge index based on five components (knowledge in nutrition, knowledge of micronutrients, label reading and numeracy, food measure skill, and grouping food in groups) and found that double burden of malnutrition was among the children of low maternal nutritional literacy households (26). Another study in urban Indonesia used maternal nutritional knowledge for obese mothers based on three dimensions such as macronutrients knowledge, household food measure skills, grouping food into categories skill and concluded that maternal knowledge has increased the self-efficacy in mothers and provided improvements in children growth behaviors (27). A study in Iran used food and nutrition knowledge assessment index using 60 items based on six dimensions such as knowledge on nutrition and food, functional skills, interactive skills, advocacy, critical investigation of information, and skills on reading a food label and concluded that nutrition knowledge level among senior-high-school students was low while academic performance and socioeconomic status were significant predictors (28).

Studies from Pakistan and India also highlighted the impact of MNHA on malnutrition. A study conducted in different areas of Pakistan (Quetta and Tandojam) used nutrition education intervention based on the counseling of mothers. The intervention was based on breastfeeding and food items and there was a significant reduction in malnutrition after this intervention (29). Another study in Pakistan developed mothers' knowledge, perceptions, and knowledge on health based on eight components awareness on child weight and height gain, food type, number of times a day child feed, knowledge on solid foods, ORS or boiled water, cleanliness, maintaining contact with lady health workers, and breastfeeding practices and found that mothers' awareness and literacy was a significant predictor of better nutritional outcomes (30).

Because a mother is the prime provider of a child's nutritional needs and care, the chances of malnutrition are reduced in mothers who have high MNHA (31–33). Study in Andhra Pradesh, India assessed the mothers' knowledge, perceptions, and attitude on malnutrition based on 23 questions on child feeding and found that only 35 percent of mothers showed a positive attitude toward child feeding and 33 percent of mothers have practiced proper feeding and nutritional knowledge (34). Another study in India assessed the knowledge and attitude of mothers regarding dietary and malnutrition prevention based on 30 multiple choice questions found that mothers with ~56 percent adequate knowledge and dietary practices helped in the prevention of malnutrition among their children (35).

Studies in Africa verified the importance of nutritional and health-related awareness of mothers with malnutrition. A study in Mozambique, East Africa designed the maternal health awareness index based on responses from mothers regarding malnutrition cases, symptoms, and screening. It found that mothers could somehow be able to screen only serve malnutrition while they could not detect the early signs of stunting and undernutrition (36). Another study in west Africa, in Niger, composed mothers' perceptions and awareness on malnutrition index based on five components mainly protein deficiency, nutrients deficiency in the body, lack of right food type, deficiency of carbohydrates, and breastfeeding, and found that mothers' awareness on malnutrition had a significant association with malnutrition (37). A study from Niger also used mother nutritional related knowledge index based on five components (such as colostrum knowledge of the mother, breastfeeding knowledge, knowledge on using ORS for diarrhea prevention, knowledge on immunization of child, knowledge on family planning) and concluded that the current mothers' knowledge on nutrition is not sufficient to reduce malnutrition (38). A study in Madagascar, East Africa, used mothers' health knowledge (focusing on knowledge of food, knowledge on nutrition and malnutrition, experiences on food, knowledge on services for malnutrition, and breastfeeding) concluded that maternal health knowledge significantly contributed to the reduction of SAM cases (39). A study in Cameroon, Central Africa used mothers' nutritional knowledge index based on nine questions related to breastfeeding, food items, pre-lacteal liquids, and use of palm cooking oil and found that only seven percent of mothers breastfeed their children according to WHO guidelines, nutritional education has improved dietary habits (40).

A study in India highlighted that HDS significantly impacted child nutrition status as deprivation in basic amenities increased the malnutrition prevalence in children (24). The HDS index in India found that more than half of truly poor households had at least one child underweight or stunted in their houses as compared to non-poor counterparts (41). Literature from Pakistan indicated that households' poor socioeconomic status or poverty was a major contributing factor in child malnutrition (2–6). With the improvement in social and economic status, families have more resources to provide their children with food and nutrition as well as proper medication in case of any disease.

It is assumed that there could be a trade-off between the working status of women and child care. If women are engaged in proper employment, then child care and development especially child feeding may be affected due to less time given to the child because of employment (42). But on the other hand, the working status of women independently contributes toward children's nutrition. Women's income increases the household's resources to buy food and nutrition and afford basic amenities of life. The results of the study show that mothers who were not engaged in any employment or job had higher odds of malnutrition among their under-five children (OR = 3.31, 95% CI: 0.31–35.69). The results of a study revealed that working women belonging to the household of the first two quintiles (poorest and poorer) of the wealth index were not contributing to the nutrition of the children while in the third quintile (medium) of wealth index, the working status of women contributed to the nutritional prestige of children in Pakistan (43). It may be concluded that women's employment should be at that level that can support the socio-economic status of the household, but on the other hand, child care should not be suffered.

The logistic results of the study in district Rahimyar Khan showed that the birth order of children 4–5 years was accompanied by lower probabilities of malnutrition (OR = 0.44, 95% CI: 0.21–0.94). However, it might not have a linear relation with the increase in birth order. It may be a reflection of fact that most of the parents were fulfilling the prime food requirements of their children because in rural areas natural food items (milk, fruits, and vegetables, etc.) are cheap and easy to access. A study in Nepal depicts that birth interval <24 months was significantly associated with severe acute malnutrition (44). Likewise, a study in Pakistan illustrated that higher birth order significantly increased the odds of stunting (45). Also, another study in Pakistan showed that the probability of child mortality decreased with greater birth intervals (46).

The food and care requirements of a child can vary with age. The study results revealed that the age of children from 25 to 36 months was associated with the higher odds of malnutrition among pre-school children (OR = 8.68, 95% CI: 2.83–26.61). As the child's age increased, the probability of malnutrition also increased. It reflects that most of the parents could not fulfill the nutrition requirements of their child with increasing age or due to bad health and water facilities children failed to recover quickly. The results of the study indicated that the ages of 13–24 and 25–36 months have a significant and positive association with child malnutrition which means children aged 0–36 months have a greater risk of malnutrition than those above 36 months. The findings in Pakistan and Bangladesh are in line with our results in which the age of the child is positively associated with child malnutrition (34, 47).

The results of ethnicity in the current study illustrated that children who belonged to Saraiki ethnicity had lower chances of becoming malnourished as compared to Punjabi counterparts (OR = 0.39, 95% CI: 0.15–0.98). The outcome reflected that in the total sample the rate of malnutrition prevalence in Punjabi's children is higher than the Saraiki children because the average total income of Punjabis was less than the average total income of Saraiki households in district Rahim Yar khan. Income could be a factor to increase malnutrition in Punjabi children. A study in Nepal regarding ethnicity shows that Dalit children were more underfed as compared to Brahmin children (48). In another study, the ethnicity variable was also significantly impacting the nutritional status of children in Vietnam (49).

## Conclusion

This study investigates the association between mothers' nutritional and health awareness and child nutritional status in a marginalized district of Punjab province of Pakistan. Results reveal that health and nutritional awareness in mothers strongly contribute to child malnutrition especially when households are socioeconomically deprived. It implies that these two factors have a syndemic relationship as they mutually reinforce each other. Therefore, both factors need to be compositely dealt with to tackle child malnutrition side by side. As this district has remained marginalized in terms of socioeconomic conditions there is a strong need to create income-generating opportunities along with social security nets to end the deprivation. Additionally, one of the best ways to raise awareness on health and nutrition might be Lady Health Workers (LHWs) program with proper training because they are often in close contact with pregnant and lactation women.

## Limitations of Study

Due to the financial and logistical limitations, the study only covered one of the most deprived districts of Punjab and restricted its sample size to 384 households.

## Data Availability Statement

The raw data supporting the conclusions of this article will be made available by the authors, without undue reservation.

## Ethics Statement

The studies involving human participants were reviewed and approved by Graduate Research Management Council (GRMC) in its 6th meeting through the Department of Health Economics at Pakistan Institute of Development Economics (PIDE). Written informed consent from the participants' legal guardian/next of kin was not required to participate in this study in accordance with the national legislation and the institutional requirements.

## Author Contributions

MS and FA designed the study and drafted the manuscript. YC, FA, SR, JG, NM, UR, MQ, RS, and RM were involved in manuscript revision. All authors have contributed equally in approving this manuscript to its final version.

## Conflict of Interest

The authors declare that the research was conducted in the absence of any commercial or financial relationships that could be construed as a potential conflict of interest.

## Publisher's Note

All claims expressed in this article are solely those of the authors and do not necessarily represent those of their affiliated organizations, or those of the publisher, the editors and the reviewers. Any product that may be evaluated in this article, or claim that may be made by its manufacturer, is not guaranteed or endorsed by the publisher.
